# Methodology of Natsal-COVID Wave 1: a large, quasi-representative survey with qualitative follow-up measuring the impact of COVID-19 on sexual and reproductive health in Britain

**DOI:** 10.12688/wellcomeopenres.16963.2

**Published:** 2022-03-28

**Authors:** Emily Dema, Andrew J Copas, Soazig Clifton, Anne Conolly, Margaret Blake, Julie Riddell, Raquel Boso Perez, Clare Tanton, Chris Bonell, Pam Sonnenberg, Catherine H Mercer, Kirstin R Mitchell, Nigel Field

**Affiliations:** 1 Institute for Global Health, University College London, Mortimer Market Centre, London, WC1E 6JB, UK; 2 NatCen Social Research, 35 Northampton Square, London, EC1V 0AX, UK; 3 Ipsos MORI, 3 Thomas More Square, London, E1W 1YW, UK; 4 MRC/CSO Social and Public Health Sciences Unit, University of Glasgow, 99 Berkeley Street, Glasgow, G3 7HR, UK; 5 Faculty of Public Health & Policy, London School of Hygiene & Tropical Medicine, Keppel Street, London, WC1E 7HT, UK

**Keywords:** COVID-19, population estimates, online survey, sexual behaviour, sexual health, relationships

## Abstract

**Background: **Britain’s National Surveys of Sexual Attitudes and Lifestyles (Natsal) have been undertaken decennially since 1990 and provide a key data source underpinning sexual and reproductive health (SRH) policy. The COVID-19 pandemic disrupted many aspects of sexual lifestyles, triggering an urgent need for population-level data on sexual behaviour, relationships, and service use at a time when gold-standard in-person, household-based surveys with probability sampling were not feasible. We designed the Natsal-COVID study to understand the impact of COVID-19 on the nation’s SRH and assessed the sample representativeness.

**Methods: **Natsal-COVID Wave 1 data collection was conducted four months (29/7-10/8/2020) after the announcement of Britain’s first national lockdown (23/03/2020). This was an online web-panel survey administered by survey research company, Ipsos MORI. Eligible participants were resident in Britain, aged 18-59 years, and the sample included a boost of those aged 18-29. Questions covered participants’ sexual behaviour, relationships, and SRH service use. Quotas and weighting were used to achieve a quasi-representative sample of the British general population. Participants meeting criteria of interest and agreeing to recontact were selected for qualitative follow-up interviews. Comparisons were made with contemporaneous national probability surveys and Natsal-3 (2010-12) to understand bias.

**Results: **6,654 participants completed the survey and 45 completed follow-up interviews. The weighted Natsal-COVID sample was similar to the general population in terms of gender, age, ethnicity, rurality, and, among sexually-active participants, numbers of sexual partners in the past year. However, the sample was more educated, contained more sexually-inexperienced people, and included more people in poorer health.

**Conclusions: **Natsal-COVID Wave 1 rapidly collected quasi-representative population data to enable evaluation of the early population-level impact of COVID-19 and lockdown measures on SRH in Britain. Although sampling was less representative than the decennial Natsals, Natsal-COVID will complement national surveillance data and Natsal-4 (planned for 2022).

## Key messages

- Sexual and reproductive health (SRH) remains important during the COVID-19 pandemic.
- However, a lack of attention to this aspect of health and well-being, as well as the challenges of addressing this sensitive topic have meant that the impacts of the pandemic for sexual behaviour and SRH have largely been ignored.
- Non-pharmaceutical interventions to reduce SARS-CoV-2 transmission meant that in-person, household-based probability methods were not feasible during this time.
- Incorporating learning from the decennial Natsal survey, Natsal-COVID demonstrates the feasibility of obtaining valuable public health data from a web-panel using quota-sampling and weighting to provide a quasi-representative national sample.
- Natsal-COVID is unique in the pragmatic collection of population-level SRH data to inform policy and practice responses to the pandemic in a timely manner and supplement data from surveillance systems, service users, and the decennial Natsal study

## Background

Sexual and reproductive health (SRH) is integral to wider health and well-being ^1^. Measuring and monitoring SRH is crucial in normal times, and remains so during the COVID-19 pandemic ^2^. However, none of the large national surveys undertaken to assess the impact of COVID-19 and associated restrictions included questions about sexual behaviour or SRH. This partly reflects the challenges of asking about sensitive and sometimes stigmatising behaviours, and also longstanding failure to prioritise this aspect of individual and public health ^3^. Furthermore, existing cohort studies in Britain do not focus on SRH, so it was not possible to use these as was done for some other areas of health (e.g., UK Household Longitudinal Study) ^4^.

The largest and most comprehensive population-based studies of SRH in Britain are the decennial National Surveys of Sexual Attitudes and Lifestyles (Natsal). These have developed rigorous methods to obtain high-quality data, including optimising sampling, data collection methods, and question wording. Consequently, findings from Natsal have informed SRH policy and practice in Britain and internationally since 1990 ^5^. However, due to the risks of COVID-19, lockdown restrictions, and the need for timely data, Natsal’s methods (i.e., household-based interviewing and probability sampling using the Postal Address Files (PAF)) were not feasible at this time. The pilot for the fourth decennial Natsal survey was paused until 2021 due to these restrictions, allowing the team to conduct the Natsal-COVID study. The Natsal-COVID study sought to capitalise on the experience of Natsal to understand the impact of COVID-19 on SRH in Britain and took a pragmatic approach to achieve the best quality possible under the circumstances. We prioritised a large-scale national quota sample with online data collection that could be achieved rapidly and at relatively low cost, which facilitates multiple waves of data collection to monitor changes during and after the pandemic. We also undertook qualitative interviews to gain greater contextual understanding and to provide insights into the thoughts, feelings, and behaviours of participants reporting three types of experience in the survey, chosen for their public health importance. The reported experiences were: sex with someone outside the household during lockdown; difficulty accessing SRH services; and relationship difficulties A second wave conducted in March-April 2021 captures behaviour and outcomes one-year after the first national lockdown in Britain (due to report in July 2021).

**Figure 1.**
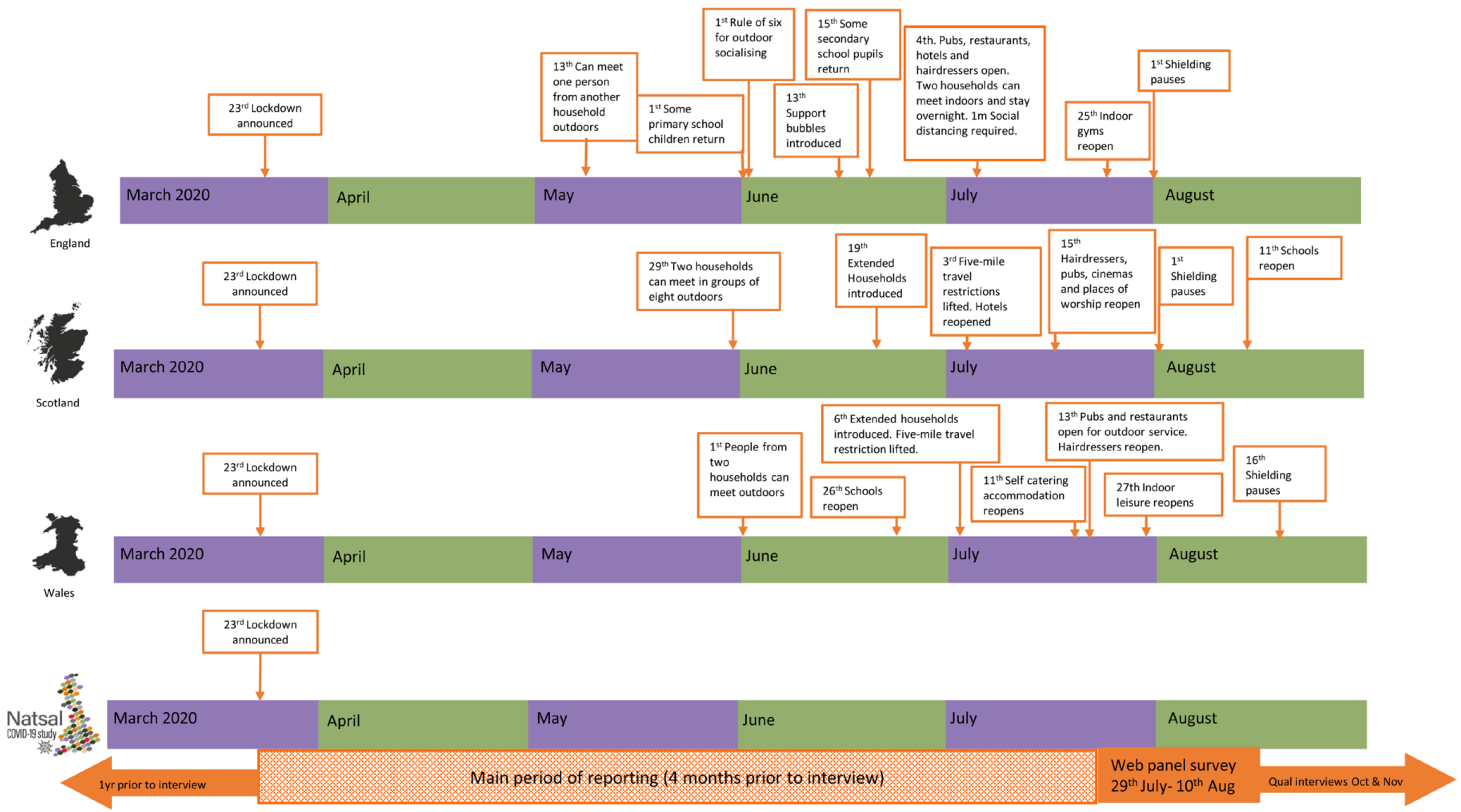
Timeline of Natsal-COVID study and COVID-19 restrictions in Britain.

In response to increasing COVID-19 infections, the UK government announced the first national lockdown on 23 March 2020, which meant individuals were asked to stay at home except for essential shopping, medical care, and exercise ^6, 7^. From mid-May, restrictions were gradually eased. However, some form of restrictions and physical distancing requirements remained throughout the summer ( Figure 1). Wave 1 of Natsal-COVID aimed to understand early changes in SRH service use and need, sexual behaviours, and relationships during this time. It was designed to capture experiences during the four months following the beginning of the first national lockdown in the UK, including a period of subsequent partial easing of restrictions ( Figure 1). This paper describes the methods used in the Wave 1 of Natsal-COVID and assesses the representativeness of the data.

## Sample design

The target sample size was primarily based on the detection of change in key behavioural and other outcomes over time between the first (Wave 1) and follow-up (Wave 2) samples with comparisons within-person based on those completing both Waves. We can also consider ‘cross-sectional’ analyses within each Wave in selecting the sample size. We set an initial sample size target of 6,000 participants aged 18–59 years old for Wave 1 and assumed that ≥2,000 of each gender (4,000 in total) would complete follow-up (those from Wave 1 who do not complete Wave 2 are ‘replaced’ by new participants for Wave 2). We assumed a design effect of <1.33 giving an effective sample size of ≥1,500 of each gender (3,000 in total) completing follow-up. To detect a 5% significance level (two-sided testing) of change over time in a less common behaviour for each gender (such as partner change), we anticipated over 80% power to detect change as small as 2 vs. 3% (RR 1.5), provided the correlation over time within individuals was moderate as expected (>0.25). For less common behaviours reported primarily by one gender (e.g., emergency contraception use), power was anticipated to be >80% for change as small as 2 vs. 3.5% (RR 1.75). For a common behaviour, such as sex in the past four weeks by gender, power was anticipated as >80% even for small changes such as 50 vs. 55% (RR 1.1), provided the correlation over time was >0.1. This sample size of 6,000 participants per Wave, assuming a design effect of <1.33, provides an effective sample size >2,200 of each gender at each Wave. For a ‘cross-sectional’ analysis of change between Waves within each gender, including all participants from both Waves and conservatively ignoring the correlation between responses from the same individuals, the design provides 90% power to detect differences in outcomes such as 2.0% vs. 3.6% and 50% vs 55%. The core sample therefore included 6,000 people aged 18–59 years with an additional 'boost sample’ of 500 people aged 18–29 years to ensure a sample of 2,000 people aged 18–29 was achieved across whole sample. The boost was added because the burden of SRH needs falls predominantly on young people.

Quotas were set for the core and boost samples based on gender, age, region, and social grade to achieve a quasi-representative sample of the general population aged 18–59 years. The quotas for gender, age, and region used ONS mid-year estimates for 2019 ^8^. The quotas for social grade used census data from 2011 (as mid-year or more recent estimates were not available for this measure) ^9^, which used data for those aged 16–59 years rather than 18–59 years due to data availability.

## Ethical approval

We obtained ethics approval from University of Glasgow MVLS College Ethics Committee (reference 20019174) and London School of Hygiene and Tropical Medicine Research Ethics committee (reference 22565). Participants provided consent to participate via an online consent form prior to the start of the survey.

## The questionnaire

The Natsal-COVID questionnaire was adapted from the Natsal-3 questionnaire ^10, 11^. and informed by development work for Natsal-4, which was paused until mid-2021 due to the pandemic. Questions specific to the COVID-19 pandemic were included, many of which were drawn from other major COVID-19 studies ^12– 14^. Some question wording was adapted for an online mode of delivery. For example, timeframes were changed, pop-up boxes were used to show definitions of key terms (e.g., oral, anal, and vaginal sex), and the survey length and complexity were reduced to improve completion without an interviewer being present. No formal validation testing was conducted for the whole questionnaire, but many questions were based on previously validated wording (e.g. Natsal-3), which was updated to reflect the time frames needed in the context of the pandemic. Some measures which were included in the Natsal-COVID questionnaire, such as the generalised anxiety disorder two item (GAD-2) and patient health questionnaire two item (PHQ-2) scales, have been described and validated elsewhere ^15, 16^.

Natsal-COVID included questions on sexual activity and relationships over different timeframes, including since the start of the first national lockdown, intimate contact with people outside of their household since lockdown, as well as relationship quality and sexual function, and SRH service use and unmet need ( Box 1). The full questionnaire is available at the study website and will be made available as *Extended data*. As in the decennial Natsal, the Natsal-COVID survey utilised routing to minimise participants being asked questions irrelevant to their own situation and experiences. In line with other COVID-19 surveys and due to the lack of a baseline immediately prior to the pandemic, the questionnaire included questions on perceived changes compared to the three months prior to lockdown. For further information about the questionnaire, interested researchers may contact the Natsal team ( natsal@ucl.ac.uk).

**Box 1. Natsal-COVID Wave 1 questionnaire content**

Gender identity, sex at birth
Who you’ve been living with since lockdown
General health and disability
COVID –19: shielding letter, diagnosis, symptoms
Alcohol consumption
Mental health -- Generalised anxiety disorder two item (GAD-2) and patient health questionnaire two item (PHQ-2) scales
Ethnicity
Sexual identity
Employment status
Education
Number of opposite-sex, same-sex, and transgender partners in different time periods (lifetime, one year, since lockdown, past four weeks)
Condomless sex with new opposite-sex, same-sex, and transgender partners in different time periods (one year, since lockdown)
Romantic or sexual experiences outside of the household (past four weeks)
Sexual behaviours since lockdown
Sexual function
Access to sexual and reproductive health (SRH) services
Unmet need for SRH services
Method of accessing sexually transmitted infection (STI) testing services
Contraception used since lockdown
Condom access since lockdown
Changes in sexual relationships since lockdown
Relationship quality since lockdown

## Sample recruitment

**Figure 2.**
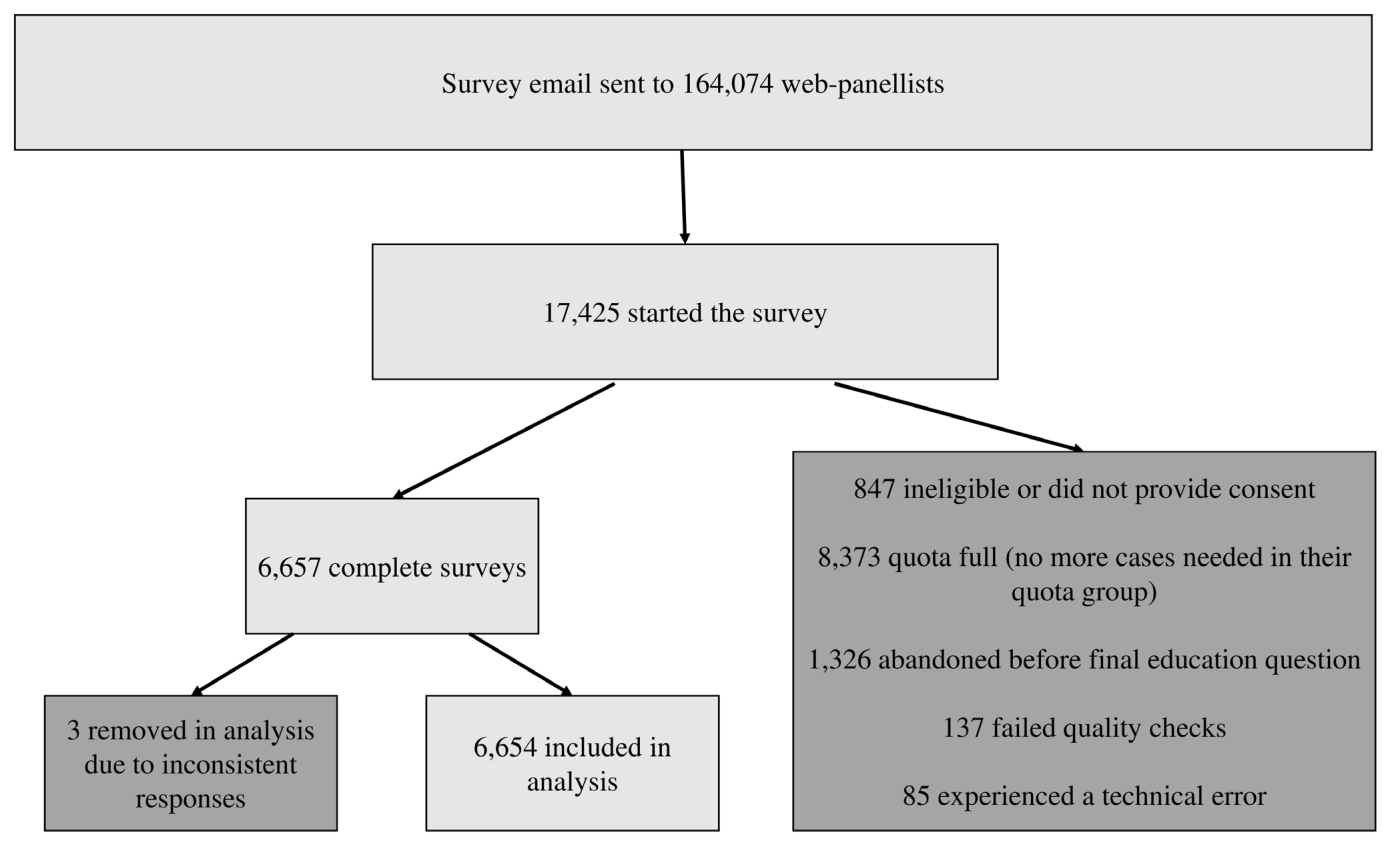
Recruitment process for Natsal-COVID.

Survey data were collected from 29 July 2020 to 10 August 2020. The online panels are run with stringent recruitment and quality-control processes to ensure individuals can only join once, are not excessively sampled for surveys, and so remain engaged. 164,074 panellists were contacted via email to participate in Natsal-COVID ( Figure 2). Of those who were emailed, 17,425 panellists started the survey, of whom 88% came from Ipsos MORI’s own panel, with ‘top up’ from six other panel providers used by Ipsos MORI. Of the 17,425 participants starting the survey and providing demographic information for the quotas, 847 were ineligible or did not provide consent, 8,373 were diverted from completing the survey because their quota group (described below) was full, 1,326 participants abandoned the survey before completion, 137 failed quality checks, and 85 experienced a technical error. Overall, 6,657 participants completed the survey. A further three participants were removed from the sample due to inconsistent responses, giving a final sample size of 6,654. Web-panel methodology precludes calculation of any response rates because panellists are invited in waves and selected based on quotas.

About half of participants completed the survey on a laptop or desktop (49%), and the rest via smartphone (45%) or tablet (6%). Median survey length was 10 minutes; the interquartile range was 7 minutes to 14 minutes.

Panellists receive small incentives to participate in Ipsos MORI surveys in the form of points, which can be redeemed for modest rewards and entry into sweepstake draws. Panellists who do not qualify for a survey (i.e., do not progress beyond the screening questions) also receive a small number of points for their willingness to participate.

## Gender in Natsal-COVID

**Figure 3.**
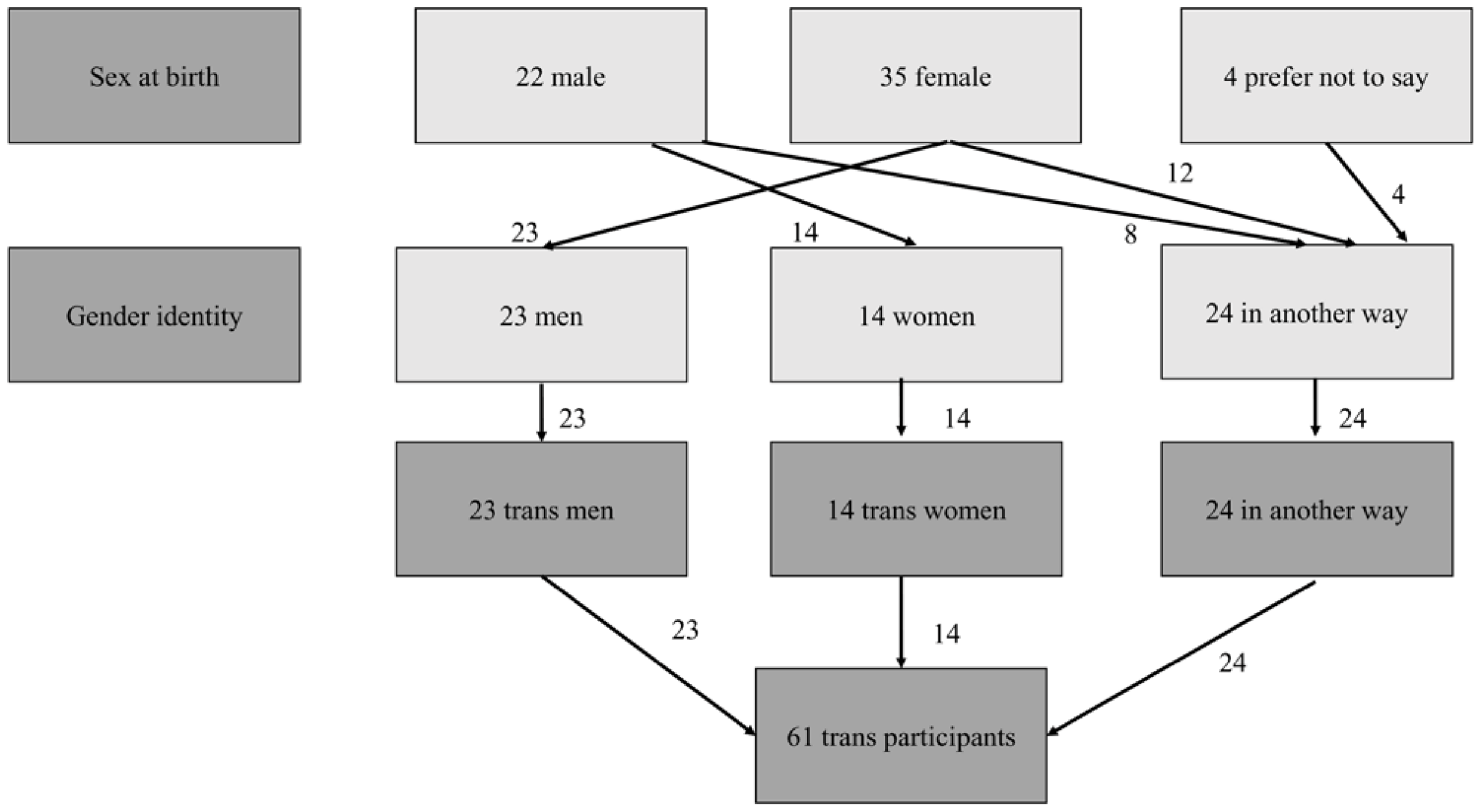
Classification of Trans participants.

Natsal-COVID was inclusive in its approach to gender, including in response options and questionnaire routing. Although panel quotas were based on the proportions of participants identifying as men and women (i.e., not including ‘in another way’), the survey asked participants to self-report gender identity, with the options being ‘man’, ‘woman’, or ’in another way’ (e.g., non-binary), and sex assigned at birth (options being ‘male’, ‘female’, or ‘prefer not to say’). Sixty-one participants were classified as 'trans’ (a derived variable) where their reported sex at birth was different to their reported gender identity, including 24 trans men, 14 trans women, and 24 who identified in another way ( Figure 3), giving an overall percentage of 0.8% in the weighted sample ( Table 1). Where data are presented for men and women, these estimates include trans men and trans women, respectively. The participants identifying ‘in another way’ were included in analyses where the denominator is everyone but were not included in denominators for men or women.

**Table 1.** Natsal-COVID unweighted and weighted distributions of quota and weighting variables compared with external probability surveys.

		Men (including Trans Men)			Women (including Trans Women)			All		
		% [95% CI]			% [95% CI]			% [95% CI]		
		**Natsal-COVID**		**Population** **estimate ^1^**	**Natsal-COVID**		**Population** **estimate ^1^**	**Natsal-COVID**		**Population** **estimate ^1^**
**Denominators** **(weighted,** unweighted) *		3310, 3187		17520655, 61993	3320, 3443		17668414, 68988	6654, 6654		35189069, 130981
		*Unweighted %*	*Weighted %* *[95% CI]*		*Unweighted %*	*Weighted % [95%* *CI]*		*Unweighted %*	*Weighted %* *[95% CI]*	
**Gender**	Men	-	-	-	-	-	-	47.9	49.8 [48.5,51.0]	49.8 [49.5,50.1]
	Women	-	-	-	-	-	-	51.7	49.9 [48.6,51.2]	50.2 [49.9,50.5]
	Trans ^2^	0.7	0.6 [0.4,0.9]	-	0.4	0.4 [0.2, 0.7]	-	0.9	0.8 [0.6,1.1]	-
**Age**	18–24	12.6	13.6 [12.3,15.0]	15.6 [15.3,16.0]	17.3	12.0 [11.1,13.1]	14.8 [14.5,15.2]	15.2	12.9 [12.1,13.8]	15.2 [15.0,15.5]
	25–34	24.3	25.5 [23.91,27.2]	25.0 [24.6,25.4]	33.3	27.6 [26.1,29.2]	24.6 [24.2,25.0]	29	26.6 [25.5,27.7]	24.8 [24.5,25.1]
	35–44	23.1	23.8 [22.3,25.5]	22.9 [22.5,23.3]	21.4	24.5 [23.0,26.1]	23.2 [22.8,23.6]	22.2	24.1 [23.0,25.3]	23.1 [22.8,23.3]
	45–59	40	37.1 [35.3,38.8]	36.5 [36.0,36.9]	28	35.89 [34.1,37.7]	37.4 [27.0,37.8]	33.7	36.4 [35.1,37.6]	36.9 [36.6,37.2]
	Median (IQR) [95 ^th^ percentile]	40 (29, 51) [58]	39 (29, 49) [58]	38 (28, 49) [57]	34 (26, 46) [58]	39 (29, 50) [58]	39 (29, 50) [57]	37 (28, 48) [58]	39 (29, 49) [58]	39 (29, 49) [57]
**Region**	England	89	86.9 [85.4,88.2]	86.9 [86.7,87.2]	88	86.6 [85.3,87.9]	86.6 [86.4,86.9]	88.5	86.7 [85.8,87.6]	86.8 [86.6,87.0]
	Wales	3.9	4.7 [3.9,5.6]	4.7 [4.6,4.8]	3.9	4.7 [3.9,5.6]	4.7 [4.6,4.8]	3.9	4.7 [4.1,5.3]	4.7 [4.6,4.8]
	Scotland	7.2	8.5 [7.4,9.7]	8.4 [8.1,8.6]	8.1	8.7 [7.7,9.8]	8.7 [8.5,8.9]	7.7	8.6 [7.9,9.4]	8.5 [8.4,8.7]
**Social** **grade ^2^**	A Upper middle class/ B Middle class	25.6	23.1 [21.6,24.6]	-	24.2	22.2 [20.8,23.7]	-	24.8	22.6 [21.6,23.7]	-
	C1 Lower middle class/C2 Skilled working class	52.6	53.0 [51.2,54.9]	-	50.9	52.4 [50.6,54.2]	-	51.7	52.7 [51.4,54.0]	-
	D Working class/E Lower level of subsistence	21.8	23.9 [22.3,25.6]	-	25	25.4 [23.9,27.0]	-	23.4	24.7 [23.5,25.8]	-
**Ethnicity**	White ^3^	89.5	85.6 [84.0,87.1]	84.7 [84.4,85.1]	89.4	85.8 [84.3,87.2]	83.8 [83.4,84.1]	89.4	85.7 [84.6,86.7]	84.3[84.0,84.5]
	Mixed/multiple ^4^	1.9	1.7 [1.3,2.2]	1.3 [1.2,1.4]	2.3	1.7 [1.3,2.2]	1.4 [1.3,1.5]	2.1	1.7 [1.4,2.0]	1.3 [1.3,1.4]
	Asian/Asian British ^5^	6.4	8.2 [7.2,9.4]	8.4 [8.2,8.7]	5.7	8.0 [6.9,9.2]	8.6 [8.4,8.9]	6.1	8.11 [7.4,8.9]	8.5 [8.3,8.7]
	Black/Black British ^6^	1.8	3.3 [2.5,4.2]	3.4 [3.3,3.6]	2.1	3.5 [2.8,4.5]	4.0 [3.8,4.2]	2	3.4 [2.8,4.0]	3.7 [3.6,3.9]
	Other	0.4	1.3 [0.7,2.2]	2.1 [2.0,2.3]	0.5	0.9 [0.6,1.5]	2.2 [2.0,2.3]	0.5	1.1 [0.8,1.6]	2.1 [2.0,2.2]
**Sexual identity**	Heterosexual/straight	86.8	96.2 [95.7,96.6]	94.4 [94.1, 94.7]	89.5	96.4 [95.9,96.8]	94.9 [94.7,95.1]	87.9	96.0 [95.6, 96.3]	94.6 [94.4, 94.8]
	Gay/Lesbian	7.9	2.4 [2.1,2.7]	1.9 [1.7,2.1]	2.2	1.1 [0.8,1.4]	0.9 [0.8,1.0]	5	1.8 [1.4,2.0]	1.4 [1.3, 1.5]
	Bisexual	4.6	0.9 [0.8,1.1]	0.6 [0.5,0.7]	7.2	1.8 [1.5,2.0]	1.1 [1.0,1.2]	6	1.4 [1.3,1.6]	0.9 [0.8, 1.0]
	Other	0.8	0.6 [0.4,0.9]	0.5 [0.4,0.6]	1.3	0.8 [0.5,1.1]	0.6 [0.5,0.7]	1.1	0.8 [0.6,1.0]	0.6 [0.5, 0.7]

CI=confidence intervals. *Data are presented for individuals aged 18-59 from England, Scotland, and Wales. Sexual identity comparisons (“don’t know” are excluded from the table but included in denominator) come from a report, which reports sexual identity for individuals aged 16+ in the entire UK. 1. Comparison data from Annual Population Survey 2019 (APS). 2. Data on social grade and trans in the external data sets (APS 2019 and HSE 2018) were not available 3. White includes all those who identify as White English, Welsh, Scottish, Northern Irish, British, Irish, Gypsy or Irish Traveller, or from any other White background 4. Mixed ethnicity includes those who identify as White and Black African, White and Black Caribbean, White and Asian, or any other mixed or multiple ethnic background 5. Asian includes those who identify as Indian, Pakistani, Bangladeshi, Chinese or from any other Asian background 6. Black includes those who identify as African, Caribbean, or from any other Black background

## Quota filling and weighting of survey data

Towards the end of fieldwork, some quotas were relaxed to ensure enough people from harder-to-recruit groups were included. Initially, regional quotas were relaxed to increase the number of young men, and over this period, the numbers in the lower social grades were also increased. Subsequently, all other quotas were relaxed to increase the number of participants in Scotland and Wales. Overall, this meant that target quotas (age, gender, region, and social grade) exceeded 90% in the final sample, with the exception of regional quotas for Wales (82%) and Greater London (87%).

Weighting was used to achieve a quasi-representative sample of the population of Britain by gender, age, region, social grade, ethnicity, and sexual identity. Weighting targets were based on ONS 2019 mid-year census estimates ^8^ for age, gender, and region and 2011 census figures ^9^ for social grade and ethnicity. The initial weights (including gender, age, region, social grade, and ethnicity) did not include sexual identity but we observed over-representation of non-heterosexual individuals (unweighted; Table 1), which we would expect to introduce bias within a study on sexual health. A final weight was created to include sexual identity, based on the Annual Population Survey (APS) 2018, which was a probability sample survey of those aged 16 years and older in the UK ^17^.

Ipsos MORI calculated weights using the random iterative method based on regression analysis (see Ipsos MORI Technical Report). The weight calculations were repeated until the weights were sufficiently close to the target for all factors; this convergence occurred on the fourth iteration. The weighting efficiency for the weights was 87.0%.

## Representativeness of the Natsal-COVID sample

The Natsal-COVID sample was compared with the following probability sample surveys to assess its representativeness ( Table 1 and Table 2): the 2019 Annual Population Survey (APS) ^18^, the 2018 Health Survey for England (HSE) ^19^ (general health and urban rural classification), the 2018 APS report on sexual identity ^17^, and the 2010-12 Natsal-3 study (sexual behaviour) ^5^. External datasets were restricted to 18-59-year-olds from England, Scotland, and Wales except where noted. When comparing Natsal-COVID with the HSE dataset, data are shown only for participants from England. The archived datasets were accessed from the UK Data Archive and Office for National Statistics (ONS) website. Data analysts (ED, SC, JR) had complete access to the datasets.

**Table 2.** Natsal-COVID distributions compared with external probability surveys and Natsal-3 data.

		Men (including Trans Men)			Women (including Trans Women)			All		
		% [95% CI]			% [95% CI]			% [95% CI]		
		**Natsal-COVID**		**Population** **estimate ^1^**	**Natsal-COVID**		**Population** **estimate ^1^**	**Natsal-COVID**		**Population** **estimate ^1^**
**Denominators (weighted,** unweighted) *		3310, 3187		17520655, 61993	3320, 3443		17668414, 68988	6654, 6654		35189069, 130981
		*Unweighted* *%*	*Weighted %* *[95% CI]*		*Unweighted* *%*	*Weighted %* *[95% CI]*		*Unweighted* *%*	*Weighted %* *[95% CI]*	
**Married**	Yes	38.7	39.8 [37.9,41.6]	46.8 [46.4,47.3]	36	41.4 [39.6,43.2]	48.2 [47.7,48.6]	37.2	40.5 [39.2,41.7]	47.5 [47.2,47.8]
**Education ^2, 3^**	No qualification	4.1	4.3 [3.5,5.1]	11.7 [10.3,13.2]	3.2	3.5 [2.8,4.3]	11 [9.7,12.3]	3.6	3.9 [3.4,4.4]	11.3 [10.4,12.3]
	Below degree	50	49.9 [48.0,51.9]	54.8 [52.5,57.1]	46.6	47.1 [45.2,49.0]	53 [51.0,54.9]	48.3	48.6 [47.2,49.9]	34.8 [33.4,36.3]
	Degree	46	45.9 [43.9,47.8]	33.5 [31.4,35.7]	50.2	49.4 [47.5,51.4]	36.1 [34.2,38.0]	48.1	47.6 [46.2,48.9]	53.9 [52.4,55.4]
**Rurality ^2^**	Urban	87	87.4 [86.0,88.7]	84.3 [82.7,85.7]	84.8	85.1 [83.6,86.5]	84 [82.6,85.3]	85.9	86.3 [85.3,87.3]	84.1 [83.1,85.1]
	Rural	13	12.6 [11.3,14.0]	15.7 [14.3,17.3]	15.2	14.9 [13.5,16.4]	16.0 [14.7,17.4]	14.1	13.7 [12.7, 14.7]	15.9 [14.9,16.9]
**General health** **status ^2^**	Good/ Very Good	73.1	74.0 [72.3,75.7]	81.3 [79.5,82.9]	73.3	72.9 [71.1,74.5]	78.6 [77.0,80.1]	73.1	73.3 [72.1,74.5]	79.9 [78.7,81.1]
	Fair	21	20.4 [18.9,22.0]	13 [11.7,14.5]	21.6	21.8 [20.3,23.4]	15.2 [13.8,16.6]	21.3	21.1 [20.0,22.3]	14.1 [13.1,15.1]
	Bad/ Very bad	5.9	5.6 [4.8,6.5]	5.7 [4.7,6.9]	5.2	5.3 [4.5,6.3]	6.2 [5.4,7.2]	5.6	5.6 [5.0,6.2]	6.0 [5.3,6.7]
**Limiting long-term** **illness/** **disability**	Yes	31.1	28.9 [27.2,30.6]	28.4 [28.0,28.8]	37	35.8 [34.1,37.6]	32.5 [32.1,32.9]	34.2	32.5 [31.3,33.7]	30.5 [30.2,30.8]
**Ever had any** **partnered sexual** **experience (not** **necessarily** **involving genital** **contact) ^4, 5^**	Yes	91	90.5 [89.3,91.6]	98.7 [98.4,99.0]	90	89.4 [88.1,90.5]	98.8 [98.4,99.1]	90.4	89.9 [89,90.6]	98.8 [98.5,99.9]
**Ever had vaginal,** **anal, oral sex** **or other genital** **contact with a** **partner of any** **gender ^4, 5^**	Yes	85.9	85.1 [83.5, 86.5]	96.6 [96.0,97.0]	83.1	82.7 [81.2, 84.2]	97.1 [96.6,97.5]	84.4	83.8 [82.7, 84.9]	96.8 [96.5,97.2]
**Ever had vaginal,** **anal, oral sex** **or other genital** **contact with** **a same sex** **partner ^4, 5^**	Yes	13.2	6.3 [5.6,7.2]	5.7 [5.0,6.5]	9.1	5.4 [4.7,6.1]	7.3 [6.7,8.0]	11	5.8 [5.3,6.4]	6.5 [6.0, 7.1]
**Partner numbers,** **lifetime ^4, 6^**	1 partner	12.6	13.1 [11.7,14.7]	11.9 [10.7, 13.1]	16.9	17.7 [16.1,19.4]	18.7 [11.5,19.9]	14.6	15.4 [14.2,16.5]	15.3 [14.5,16.2]
	2 partners	8.9	9.9 [8.6,11.3]	7.9 [6.9,8.9]	12.3	12.0 [10.7,13.5]	10.1 [9.2,10.9]	10.6	10.9 [10.0,11.9]	8.9 [8.3,9.6]
	3–4 partners	14.5	15.7 [14.1,17.3]	14.8 [13.6,16.1]	17.5	17.9 [16.3,19.6]	19.4 [18.3,20.6]	16.1	16.7 [15.6,17.9]	17.1 [16.3,18.0]
	5–9 partners	23.3	23.6 [21.9,25.5]	25.6 [24.2,27.2]	24.5	24.7 [22.9,26.6]	27.1 [25.9,28.4]	23.8	24.2 [22.9,25.5]	26.4 [25.4,27.4]
	10+ partners	40.7	37.7 [35.7,39.8]	39.8 [38.1,41.5]	28.9	27.8 [25.9,29.7]	24.8 [23.6,26.0]	34.7	32.8 [31.4,34.3]	32.2 [31.2,33.3]
	Median (IQR) [95th percentile]	7 (3,15) [60]	6 (3,15) [50]	7 (3,15) [50]	5 (2,10) [30)	5 (2,10) [25]	5 (2,10) [27]	5 (2,12) [42]	5 (2,11) [37]	6 (3,12) [35]
**Ever had vaginal,** **anal, oral sex** **or other genital** **contact with** **partner of any** **gender in the last** **year ^4, 5^**	Yes	70.8	70.3 [68.4,72.1]	92.8 [91.9,93.6]	70.4	68.2 [66.3,70.0]	90.3 [89.4,91.1]	70.5	69.2 [67.8,70.5]	91.5 [90.9,92.1]
**Partner numbers,** **last year ^4, 7^**	1 partner	79.1	81.8 [79.8,83.6]	79.8 [78.4,81.2]	87.8	90.4 [89.1,91.6]	86.7 [85.7,87.6]	83.5	85.9 [84.7,87.0]	83.2 [82.4,84.1]
	2 partners	9.2	9.0 [7.7,10.5]	9.1 [8.2,10.2]	6.1	5.2 [4.3,6.3]	6.6 [6.0,7.3]	7.7	7.2 [6.4,8.1]	7.9 [7.3,8.5]
	3–4 partners	4.9	4.1 [3.2,5.2]	6.4 [5.7,7.2]	3.5	2.5 [1.9,3.2]	4.1 [3.6,4.6]	4.2	3.3 [2.8,4.0]	5.2 [4.8,5.7]
	5+ partners	6.8	5.1 [4.2, 6.3]	4.7 [4.1,5.4]	2.6	1.8 [1.4,2.5]	2.6 [2.2,3.1]	4.7	3.5 [3.0,4.2]	3.6 [3.3, 4.1]
	Median (IQR) [95th percentile]	1 (1,1) [5]	1 (1,1) [5]	1 (1,2) [5]	1 (1,1) [3]	1 (1,1) [2]	1 (1,1) [4]	1 (1,1) [4]	1 (1,1) [4]	1 (1,1) [4]
**1 or more new** **partner(s), last** **year ^4, 7^**	Yes	31.1	30.0 [27.8,32.3]	25.8 [24.3,27.3]	22.2	18.6 [16.9,20.4]	19.7 [18.6,20.8]	36.4	24.4 [23.0,25.9]	22.7 [21.8,23.7]

CI=confidence intervals. *Data are presented for individuals aged 18-59 from England, Scotland, and Wales. Sexual identity comparisons (“don’t know” are excluded from the table but included in denominator) come from a report, which reports sexual identity for individuals aged 16+ in the entire UK in 2018. 1. Comparison data from Annual Population Survey 2019 (APS) unless otherwise stated. 2. Comparison data from Health Survey for England 2018 (HSE, England only) 3. Natsal-COVID participants chose an option from the following list: (1) primary school, (2) secondary school (age under 15 years old), (3) GNVQ / GSVQ / GCSE/ SCE standard, (4) NVQ1/NVQ2, (5) NVQ3/ SCE Higher Grade/ Advanced GNVQ/ GCE A/AS or similar, (6) NVQ4 / HNC / HND / Bachelor's degree or similar, and (7) NVQ5 or post-graduate diploma. A 3-category education variable, based on a variable used by 2018 Health Survey for England (HSE), including “no qualification,” “below degree”, and “degree” was derived using the following: No qualification: 1–2; Below degree: 3–5; Degree: 6–7. 4. Comparison data from Natsal-3 5. All respondents 6. All respondents reporting at least one sexual partner in their lifetime (vaginal, anal, oral sex, or other genital contact) 7. All respondents reporting at least one sexual partner in the past year (vaginal, anal, oral sex, or other genital contact)

As expected, due to quota sampling and weighting, the Natsal-COVID sample was similar to the external datasets for gender, age, region, ethnicity, and sexual identity ( Table 1). Data on social grade in the external datasets (APS and HSE) were not available. Non-heterosexual-identifying participants were over-represented in the Natsal-COVID unweighted sample (men, 13.3%; women, 10.7%) but the weighted proportions of non-heterosexual participants were broadly comparable between Natsal-COVID (men, 3.9%; women, 3.7%) and APS (men, 3.0%; women, 2.6%) (APS data includes participants aged 16+, with no upper age limit, so differences in non-heterosexual identities between unweighted samples may be partially explained by the upper age limit of 59 years in the Natsal-COVID sample).

The weighted Natsal-COVID sample was similar to APS/HSE for other demographic variables, including urban rural classification ( Table 2). The Natsal-COVID sample under-represented participants reporting their legal marital status as being ‘married’ (40.5%) compared to 2019 APS (47.5%) and over-represented those in poorer health compared to the 2018 HSE sample (fewer Natsal-COVID participants reported ‘very good’ health, and more reported ‘fair’ health). However, there were no differences in the proportion reporting a limiting long-term illness or disability.

The Natsal-COVID sample also had a higher education level than the HSE sample ( Table 2). Only 4.3% of Natsal-COVID participants reported no qualification, compared to 11.3% of HSE participants, though this should be interpreted with caution given differences in response options between the two surveys.

The Natsal-COVID sample included a higher proportion reporting no previous sexual experience (not necessarily involving genital contact) (9.5%) compared with Natsal-3 (1.3%) ( Table 2). Natsal-COVID also had a much smaller proportion reporting sex in the past year (i.e., sexually-active) (69.2%) compared to Natsal-3 (91.5%). However, when restricted to sexually-experienced participants (at least one partner, lifetime), the distribution of partner numbers (of any gender) over their lifetime was similar between Natsal-3 and Natsal-COVID. Men in Natsal-COVID (6.3%) were equally likely to report any previous same-sex experience as men in Natsal-3 (5.7%). Women in Natsal-COVID (5.4%) were slightly less likely to report any previous same-sex experience compared to those in Natsal-3 (7.3%). The proportion of sexually-active men reporting at least one new partner in the past year was higher among the Natsal-COVID sample (30.0%) compared to Natsal-3 (25.8%). This proportion was similar among women in Natsal-COVID (18.6%) and Natsal-3 (19.7%).

## Qualitative follow-up

Semi-structured qualitative follow-up interviews were conducted with 45 selected Natsal-COVID participants to explore three types of experience reported in the quantitative survey, chosen for their public health importance: (group 1) sexual contact with someone living outside their household, (group 2) needing, but being unable to access SRH services, and (group 3) increased arguments and reduced support from their partner since lockdown. Interviewees were selected from the 771 survey participants who had agreed to follow up, provided contact details, and met the criteria of reporting one (or more) of these experiences. We used qualitative purposive sampling to: ensure variation by age, gender, ethnicity, and region; select for attributes of interest (e.g., oversampling of women in group two to reflect higher use of SRH services compared to men); and maximise information (e.g., selecting individuals who met more than one criterion).

The research team contacted, by phone or email, 143 individuals who had expressed willingness to take part in further research with information on the qualitative study and to confirm their eligibility. Of these, 67 individuals were uncontactable (either due to no response after three attempts or an incorrect phone number), 20 declined to take part, six were not eligible, two were interested but were not able to make an interview time, and three expressed interest, but the quota had already been filled. Interested participants were emailed the study documentation and given time to decide if they would like to take part. Interviews were conducted between October and November 2020. Participants were offered interview by phone or video, with all but two participants opting for phone interviews. Participants completed and returned a consent form via email, which was followed up with an audio recording prior to the start of the interview where participants verbally confirmed their consent to take part.

Interviews lasting between 36 to 92 minutes (mean duration 65 minutes) were conducted by three trained qualitative interviewers (DR, KJM, RBP). Fieldnotes reflecting on interview content were documented after each interview. Participants were offered a £30 e-voucher token of appreciation. Interview guides (to be made available as *Extended data*) covered socio-demographic characteristics, lockdown household composition, views on COVID-19 and social restriction measures, and impact of COVID-19 on personal life, in addition to questions exploring the three topics outlined above ( Box 2). Audio recordings were transcribed intelligent verbatim (transcriber discretion to omit utterances that don’t add any meaning) by transcription agencies under contract. Transcripts were reviewed by the study team to check for accuracy as well as to develop familiarity with participant accounts. Transcripts were anonymised and entered into Nvivo 12 ^20^. Data were analysed thematically using a framework approach ^21^. An individual researcher led the analysis for each of the three groups but the coding frameworks, coding decisions, and emerging themes were discussed between analysts during regular analysis meetings. The analytical approach will be described in each paper reporting the results.

**Box 2. Natsal-COVID Wave 1 qualitative interview content**

GROUP ONE: Reported sexual contact with someone living outside of their household
  Circumstances and motivations for sex with someone outside their household
  Balancing needs and risks (social, sexual, and COVID-19)
  Impression management: (not) communicating sexual encounters to others
GROUP TWO: Reported unmet sexual and reproductive health (SRH) needs
  Experiences of attempting to access, and navigating services
  Consequences of unmet SRH care needs on participants
  Attitudes to telemedicine
  Views on how SRH services could be better prepared to adapt to future pandemics
GROUP THREE: Reported increased arguments and reduced support from their partner since lockdown
  Context, and history of participants’ relationship
  Emotion-focused exploration of the dynamics of the relationship since lockdown
  Stress, coping mechanisms and impacts on other aspects of life
  Potential for relationship dissolution and expectations for the future

## Discussion

Natsal-COVID is a large, national study that was rapidly undertaken in Britain at a time when data were urgently required to understand the impact of the pandemic for SRH clinical and public health policy and decision-making. Due to restrictions on movement and meeting indoors, this was also at a time when in-person household-based probability sample surveys were not feasible nor sufficiently rapid. Natsal-COVID fills an important evidence gap because other COVID-19 studies have not addressed the population-level impact of COVID-19 on sexual behaviour and SRH, or conducted qualitative interviews to more thoroughly understand SRH challenges in the general population. The Natsal-COVID study also benefits from methodological elements developed by the Natsal team in consultation with stakeholders ^22^ over several decades to inform the most rigorous and ethical approaches when asking about highly sensitive behaviours and experiences. We have demonstrated that it is possible to achieve a large-scale quasi-representative sample within 12 days of fieldwork during a period of intense social disruption.

Natsal-COVID is not a probability sample and is therefore not truly representative of the general population ^23^. Instead, we relied upon quota-based sampling and weighting to achieve a web-panel sample that is quasi-representative of the general population in Britain in terms of age, gender, region, social grade, and sexual identity. There are well described sources of bias in web-panel surveys ^24, 25^, which might affect the results and interpretation. Previous studies have demonstrated that non-probability web-panel surveys, such as Natsal-COVID, are less representative of the general population than probability surveys, such as the decennial Natsal survey ^24^. One important source of bias in web-panels is that they include only those with access to the internet. According to an Ofcom report, although 87% of the UK population older than 16 years reported using the internet in 2019 ^26^, those in lower social grades and older adults were less likely to do so. Therefore, among our sample age range of 18-59 years, we anticipate a higher proportion of individuals with internet access.

Although quota sampling is likely to be more representative of the population than self-selecting or convenience samples ^27^, which have been the primary methods used to study sexual health in the COVID-19 pandemic, there are also limitations in the use of quota sampling for the Natsal-COVID study. Quota samples and web-panel samples are particularly susceptible to non-response and residual bias due to self-selection ^27^. Comparisons with external probability surveys and with Natsal-3 show the Natsal-COVID sample to be generally similar for key sociodemographic characteristics and sexual behaviours. However, we demonstrated appreciable sampling bias for several important characteristics that remained after applying weighting. Natsal-COVID over-represented individuals with higher education levels, which is consistent with previous findings for web samples ^23– 25^. Although Natsal-COVID had a higher proportion of participants reporting ’fair health’ compared to HSE, differences here might be due to the impact of the pandemic on perceived health status, and it was noteworthy that there were not major differences in the proportions reporting limiting long-term illness or disability. Natsal-COVID also had fewer participants reporting sex in their lifetime than we would expect from Natsal-3 (albeit that Natsal-3 was undertaken ten years ago). This could be due to differences in the question wording and participants’ understanding, a mode effect (i.e., online versus in-person), or sampling bias, and Natsal-COVID may therefore include more people at lower risk of adverse SRH outcomes (e.g., people with no previous sexual experience). Prior to weighting, Natsal-COVID had more men identifying as non-heterosexual men compared to 2019 APS. The finding of over-representation of non-heterosexual men in Natsal-COVID (prior to weighting) is consistent with previous web-panel surveys ^24^. However, weighted percentages of non-heterosexual men were comparable between Natsal-COVID and 2019 APS ^28^. So, while the findings of Natsal-COVID are likely to be broadly generalisable, its prevalence estimates should be treated with particular caution.

The inclusion of semi-structured interviews facilitated understanding of phenomena of interest from the perspective of participants. Understanding the meaning of experiences and context in which they take place, can facilitate interpretation of associations identified in quantitative data, as well as surfacing key issues not asked about in the survey ^29^. Recruitment of participants from the survey sample has several benefits including being able to identify individuals with very specific experiences of interest. The large sample frame also enabled sufficient variation on key characteristics such age, gender, and region ^30^. We were unable to triangulate across survey and interview responses due to regulations stipulating sharing of only de-identified data by Ipsos MORI. The fact that interviews were subsequent to the survey allowed participants time for reflection, and this may facilitate more candid reporting ^31^. Building rapport is also important to enabling detailed and candid accounts. It was a drawback that we were unable to conduct in-person interviews due to pandemic restrictions. Although we anticipated that most participants would opt for video interviews (in order to see the interviewer), most actually preferred to use the telephone. We did not ask participants directly, but it seems likely that this was due to privacy concerns.

In conclusion, the Natsal-COVID Wave 1 study can be considered quasi-representative of the British population, and its data can be used to quantify and better understand the initial impacts of the COVID-19 pandemic on sexual behaviour and SRH in Britain following the start of the first national lockdown in the UK in March 2020. A second Wave of data collection will enable us to capture impacts throughout the year following the first national lockdown in the UK. Although not as representative as the decennial Natsal study, the data from the Natsal-COVID study are already informing SRH policy and service delivery during and after the COVID-19 pandemic.

## Data availability

Datasets (Natsal-COVID, 2019 Annual Population Survey, 2018 Health Survey for England and 2010-12 Natsal-3 study) used in this analysis are publicly available via the UK Data Archive. Datasets can be accessed through registration with the UK Data Service.

UK Data Service: National Survey of Sexual Attitudes and Lifestyles COVID-19 Study, 2020. http://doi.org/10.5255/UKDA-SN-8865-1.

UK Data Service: Annual Population Survey, January - December, 2019. http://doi.org/10.5255/UKDA-SN-8632-4 ^32^.

UK Data Service: Health Survey for England, 2018. http://doi.org/10.5255/UKDA-SN-8649-1 ^33^.

UK Data Service: National Survey of Sexual Attitudes and Lifestyles, 2010-2012. http://doi.org/10.5255/UKDA-SN-7799-2 ^34^.

The published 2018 Annual Population Survey sexual identity tables are available as a downloadable Excel file on the ONS website.
