# Supplementary material for: Methodology of Natsal-COVID Wave 1: a large, quasi-representative survey with qualitative follow-up measuring the impact of COVID-19 on sexual and reproductive health in Britain
Source: Wellcome Open Res. 2022 Mar 28;6:209. Originally published 2021 Aug 16. [Version 2] doi: 10.12688/wellcomeopenres.16963.2 (PMC9020538; doi:10.12688/wellcomeopenres.16963.2)
Supplement: Peer review history

# Peer review: Reviewer response for version 2

**DOI:** 10.21956/wellcomeopenres.19691.r49525

**Published:** 2022-04-19

**Authors:** Jamie Frankis

**Affiliations:** 1 Department of Health & Community Sciences, School of Health and Life Sciences, Glasgow Caledonian University, Glasgow, UK

The authors have carefully addressed all of my first review comments and either provided a clear rationale, appropriate rebuttal or changed the manuscript appropriately. I therefore recommend that this important article is accepted with no further changes required.

Is the rationale for developing the new method (or application) clearly explained?

Yes

Is the description of the method technically sound?

Yes

Are the conclusions about the method and its performance adequately supported by the findings presented in the article?

Partly

If any results are presented, are all the source data underlying the results available to ensure full reproducibility?

No source data required

Are sufficient details provided to allow replication of the method development and its use by others?

Yes

Reviewer Expertise:

Sexual health, health psychology, sexual behaviour research, cross sectional longitudinal surveys, online & real world survey methodologies

I confirm that I have read this submission and believe that I have an appropriate level of expertise to confirm that it is of an acceptable scientific standard.

# Peer review: Reviewer response for version 1

**DOI:** 10.21956/wellcomeopenres.18723.r47331

**Published:** 2022-02-11

**Authors:** Laura D Lindberg

**Affiliations:** 1 Guttmacher Institute, New York, New York, USA

This study provides a technical report for the design, methodology and implementation of an internet-based survey conducted as part of Natsal (National Survey of Sexual Atittudes and Lifestyles).  Because of COVID-19, Natasl could not conduct fieldwork as usual but did a high-quality pivot to online data collection. Unlike in the United States where fielding of the parallel National Survey of Family Growth did not occur during COVID, this new NATSAL data offers the potential for gaining important insights into the impact of the pandemic on sexual and reproductive health and well-being.

 Data are not presently public available at the time of this publication; if they are now, this could be updated. 

 While I think this is an excellent manuscript that will be valuable to many readers, I have a few suggestions for improvements. These include the following:

1. Box 1- is it correct that no information about pregnancy desires, experiences, or outcomes was collected? If so, why not?
2. Explain why those under age 18 were not included.
3. Please provide citations for this assertion "Web-panel methodology precludes calculation of any response rates because panelists are invited in waves and selected based on quotas." AAPOR provides guidance for this so I'm not sure it's a correct statement or needs further clarification.
4. The difference between this sample and other national studies in the proportion reporting no previous sexual experience is striking and I feel needs more discussion/investigation. Is this universal or an issue for specific subgroups? How did your question wording compare? Are there reasons to think this could represent some real social change over time?
5. Please say more about why so many respondents in the qualitative study were uncontactable. 
6. This new paper is making waves and may be worth reflecting on. Bradley, V.C., Kuriwaki, S., Isakov, M., Sejdinovic, D., Meng, X.L. and Flaxman, S., 2021. Unrepresentative big surveys significantly overestimated US vaccine uptake. *Nature*, *600*(7890), pp.695-700. ^1^
7. I don't understand why you couldn't connect people's qual and survey interviews? Why could Ipsos MORI connect them and then not share the identifying information? This seems like a missed opportunity. 
8. Could point to other studies that also shifted to video or phone interviews during COVID. This is a new and exciting methodology that Natsal is one example of. 
9. In the conclusion, please add more detailed information on how you expect the data to be used-- what questions do you hope this data will help to answer? Also, the first sentence of the final paragraph states as fact what I think is hope -- that his study WILL 'enable us to quantify and better understand...":

Is the rationale for developing the new method (or application) clearly explained?

Yes

Is the description of the method technically sound?

Yes

Are the conclusions about the method and its performance adequately supported by the findings presented in the article?

Yes

If any results are presented, are all the source data underlying the results available to ensure full reproducibility?

Partly

Are sufficient details provided to allow replication of the method development and its use by others?

Partly

Reviewer Expertise:

survey methodology, sexual and reproductive health measurement

I confirm that I have read this submission and believe that I have an appropriate level of expertise to confirm that it is of an acceptable scientific standard.

## References

[1] : Unrepresentative big surveys significantly overestimated US vaccine uptake. *Nature* .600(7890) : 10.1038/s41586-021-04198-4 695-700 10.1038/s41586-021-04198-4 34880504PMC8653636

# Response

**Published:** 2022-03-15

**Authors:** Emily Dema

**Affiliations:** University College London, UK

*Responses are italicised.*

 This study provides a technical report for the design, methodology and implementation of an internet-based survey conducted as part of Natsal (National Survey of Sexual Atittudes and Lifestyles).  Because of COVID-19, Natasl could not conduct fieldwork as usual but did a high-quality pivot to online data collection. Unlike in the United States where fielding of the parallel National Survey of Family Growth did not occur during COVID, this new NATSAL data offers the potential for gaining important insights into the impact of the pandemic on sexual and reproductive health and well-being.

*RESPONSE: Thank you for your helpful comments, which we have addressed below. We feel these have really helped to improve the paper.*

 Data are not presently public available at the time of this publication; if they are now, this could be updated.  

*RESPONSE: Data are now available, and the Data availability statement has been updated accordingly.*

 While I think this is an excellent manuscript that will be valuable to many readers, I have a few suggestions for improvements. These include the following: 

1. Box 1- is it correct that no information about pregnancy desires, experiences, or outcomes was collected? If so, why not? 

*RESPONSE: In this first wave of the Natsal-COVID study, data on pregnancy desires, experiences, or outcomes were not collected due to the short reporting timeframe (4 months from the start of the first British lockdown). The second wave of Natsal-COVID (methods of which will be reported separately) does include questions about pregnancy outcomes and pregnancy planning.*

  

1. Explain why those under age 18 were not included. 

*RESPONSE: Those under age 18 were not included due to ethical considerations and the online nature of the survey.*

  

1. Please provide citations for this assertion "Web-panel methodology precludes calculation of any response rates because panelists are invited in waves and selected based on quotas." AAPOR provides guidance for this so I'm not sure it's a correct statement or needs further clarification. 

*RESPONSE: AAPOR provide guidance for reporting precision in non-probability samples, and has guidance for reporting response rates in probability web surveys, but not response rates for non-probability surveys. Elsewhere, some have suggested ‘metrics’ for non-probability web surveys (https://doi.org/10.1093/poq/nfn065). Taken together, we prefer to retain the sentence as written.*

  

1. The difference between this sample and other national studies in the proportion reporting no previous sexual experience is striking and I feel needs more discussion/investigation. Is this universal or an issue for specific subgroups? How did your question wording compare? Are there reasons to think this could represent some real social change over time? 

*RESPONSE: Sexual experience was determined based on reported numbers of partners in participants’ lifetime. Question wording was similar between Natsal-3 and Natsal-COVID. We also note that lifetime partner numbers were similar between Natsal-COVID and Natsal-3 when restricted to sexually-experienced participants. However, the studies are separated in time by over 10 years, and we are unable to account for secular changes in sexual behaviours that may have occurred in this time. It is therefore unclear whether this reflects a true population-level change, including due to the pandemic, is due to differences in the methodologies, or a combination of these factors. Natsal-4, starting fieldwork in late 2022, is well-placed to given further insight into these questions. We have not made further alterations to the paper. *

1. Please say more about why so many respondents in the qualitative study were uncontactable.  

*RESPONSE: For ethical reasons we set a maximum number of contact attempts at 3. Of those who were uncontactable, half were not reached within three attempts and for the other half, the phone number was incorrect. We have updated the text to include this information.*

  

1. This new paper is making waves and may be worth reflecting on. Bradley, V.C., Kuriwaki, S., Isakov, M., Sejdinovic, D., Meng, X.L. and Flaxman, S., 2021. Unrepresentative big surveys significantly overestimated US vaccine uptake. Nature, 600(7890), pp.695-700. 1 

*RESPONSE: Thank you for flagging this paper to us. We were really interested to read it. Although this provides insight into the quality of different online survey methodologies, we note that it only applies to one outcome (vaccine uptake). We take from it that the Ipsos MORI approach is better than convenience sampling. However, the complex and sometimes sensitive information collected in Natsal-COVID limits how much we can infer for our study. Nevertheless, we’ve cited this paper in our discussion.*

  

1. I don't understand why you couldn't connect people's qual and survey interviews? Why could Ipsos MORI connect them and then not share the identifying information? This seems like a missed opportunity.  

*RESPONSE: Apologies that this was not clear. In fact, we were able to connect people’s qual and quant survey data, and we have modified the paper to emphasize that this was possible.*

  

1. Could point to other studies that also shifted to video or phone interviews during COVID. This is a new and exciting methodology that Natsal is one example of.  

*RESPONSE: The Natsal-4 study is indeed using a combination of face-to-face and remote interviews. However, the Natsal-COVID quantitative survey was delivered solely online. We will report on the Natsal-4 experiences in other methodological papers in due course.*

  

1. In the conclusion, please add more detailed information on how you expect the data to be used-- what questions do you hope this data will help to answer? Also, the first sentence of the final paragraph states as fact what I think is hope -- that his study WILL 'enable us to quantify and better understand...": 

*RESPONSE: Thanks for this comment. We have modified the discussion to express this as an aspiration. The many and varied uses of the Natsal-COVID data make it difficult to list every way in which the data might be used. *

- Is the rationale for developing the new method (or application) clearly explained? 
- Yes 
- Is the description of the method technically sound? 
- Yes 

- Are sufficient details provided to allow replication of the method development and its use by others? 
- Partly 
- If any results are presented, are all the source data underlying the results available to ensure full reproducibility? 
- Partly 
- Are the conclusions about the method and its performance adequately supported by the findings presented in the article? 

- Yes 

# Peer review: Reviewer response for version 1

**DOI:** 10.21956/wellcomeopenres.18723.r45685

**Published:** 2021-09-20

**Authors:** Jamie Frankis

**Affiliations:** 1 Department of Health & Community Sciences, School of Health and Life Sciences, Glasgow Caledonian University, Glasgow, UK

**Approval Status:**

 The methods used are clearly and appropriately described within the paper and Wellcome Open Research is an appropriate forum for this paper. Accordingly, I approve this paper subject to very minor revisions, which I have detailed below.

**Article Summary:**

 This study reports the methodology, quota sampling and weighting for an internet panel-based iteration of the decennial national probability Natsal (National Survey of Sexual Attitudes and Lifestyles) study, itself the world-leading study of sexual behaviours for a national population. C19 restrictions prevented the normal sampling methodology of Natsal during 2020; this paper critically discusses the new methodology developed to address this.

**Specific questions for Article type:**

- Is the rationale for developing the new method (or application) clearly explained?

Yes. This study reports the methodology, quota sampling and weighting for an internet panel-based iteration of the national probability Natsal study, itself the world-leading study of sexual behaviours for a national population. C19 restrictions prevented the normal sampling methodology of Natsal during 2020, so this paper is required to explain the new methodology developed. Moreover, this methodology represents the state of the art in terms of developing potentially representative national samples during C19, and so the rationale for developing this method is clear. It is also directly relevant to other situations where researchers need to assess the generalisability of their internet-based samples to the wider population.

- Is the description of the method technically sound?

Yes. The authors have provided extensive details around their method which are technically sound throughout and provide useful and clear critical reflection on their achievements and limitations.

- Are sufficient details provided to allow replication of the method development and its use by others?

Yes. The authors have provided a clear explanation of the methods they have used to develop their study, achieving an optimal balance of detail and brevity where appropriate, including referring the reader to existing documentation already available to enhance their description. There are some areas where a replicator may need additional details from the authors (e.g. the developmental history of specific survey questions) but these are best obtained from directly contacting the authors, rather than including all possible permutations in the paper.

- If any results are presented, are all the source data underlying the results available to ensure full reproducibility?

N/A. Study results are not presented other than the number of types of participants, by relevant characteristics, required to generate weighted data and check the representativeness of their samples against external datasets.

- Are the conclusions about the method and its performance adequately supported by the findings presented in the article?

Yes, all conclusions are highly appropriate, except for the final conclusion, which I have commented on below. In addition, I have made a few extra comments that the authors might find useful in enhancing their paper for clarity.

**Peer Review Report:**

**Abstract:**

- It is unclear why qual participants were included so it would be helpful to briefly indicate the reason for this.No further comments by my side
- ‘and inform policy’ – this paper, which focuses on reporting study methodology, does not provide any evidence about this issue so perhaps it should be deleted from this abstract.

**Key messages:**

 These are clear and appropriate.

**Background:**

- A clear, balanced rationale has been provided for the importance of the Natsal-COVID study.
- It would be helpful to specify the dates of wave 2 and the ‘types of experience’ used to sample qual participants. It would be useful to update on the report which was due in July 2021, if possible.
- Figure 1 is helpful, but the ‘Natsal’ row needs greater explanation. Specifically, it is not immediately unclear what ‘to 1 yr prior to interview’ and ‘period of reporting’ mean.
- It is important to clarify early on that Natsal 4 was planned for 2020 but paused due to the pandemic – perhaps in paragraph 2 of the background – which is why Natsal-COVID was conducted (if this is indeed the case).

**Sample Design:**

- A clear description of the target sample size, using design effect, power, change detected and risk ratio is provided for the overall sample size and the 18-29 boost sample. Although the process described is logical, I always think it is useful to refer readers to relevant texts/papers to confirm the literature underpinning these analyses (although I note that it *is* usual to miss this out).
- It might be helpful to clarify that the 2011 census is the most recently available in the UK at this time.
- Is there a typo in the first sentence ‘and McNemar’s test’

**Ethics:**

- These issues are clearly explained. Ethics obtained from U of G and LSHTM and therefore appropriate.

**The Questionnaire:**

- Content is clearly summarized, with a convincing rationale. A good balance between brevity and depth has been achieved here and the actual survey is available online. It is always desirable for specific details of all survey questions’ development/origins to be available (and herein the C19 questions in particular). However, it is not feasible to provide such details for every item for such a large, detailed questionnaire. Therefore I agree with the level of detail provided herein and that further details should be restricted to future publications, where necessary. Perhaps the authors *could* suggest that further details about the origins/development of specific items could be provided upon request?

**Sample recruitment:**

- It would be helpful to modify the 2 ^nd^ and 3 ^rd^ sentences to clarify what the ‘online panels’ are; it is not actually made clear for folk who are not familiar with Ipsos MORI. It would be useful to know what/who the ‘six other panel providers’ were, for completeness.
- ‘Quota group’ – it might be helpful to signal that this issue is discussed in later in the paper.
- ‘Median interview length’ – it would be helpful to replace the phrase ‘interview’ here with ‘survey’ (e.g. survey completion time’) as here I assume the authors are referring to the quant survey data completion and not the later qualitative interviews? Referring to both as interviews is likely to confuse these two different parts of the data collection.

**Gender:**

- Gender has been dealt with in an appropriate way herein, and clearly explained which is commendable. In addition, a reasonable proportion of trans folk have been included in the study which is a good achievement for this kind of general population survey.

**Quota Filling:**

- It is important to have a couple of sentences to clarify what quotas were originally set up for the study as this is not clearly mentioned earlier. It might be useful to reference the technical reports mentioned later to help with this. Relaxation of quota sampling is clearly explained as is weighting.

**Representativeness:**

- This issue is clearly explained, and appropriately and comprehensively conducted with reference to appropriate external datasets. Over-representation of LGBTQI+ folk is to be expected given the focus on sexual health and sampling frame, though it *might* be worth clarifying why this is in the paper. The over-representation of folk reporting ‘fair’ as opposed to ‘very good’ health is to be expected during a global pandemic as are lower proportions reporting ‘sex in the past year’ after 4 months of lockdown and prohibited extra-domestic liaisons. Overall, a clear case is made for the representativeness of the sample given the potential for real behavioural changes engendered by the pandemic itself.

**Qualitative sampling:**

- It might be useful to clearly locate this quota sampling as qualitative purposive quota sampling in the text.
- ‘women in group 2’ – I do not understand what being in ‘Group 2’ means? This appears in Box 2 but at this stage the reader has yet to be introduced to this information. Perhaps the first sentence of this section needs to clarify this.

**Qualitative analysis:**

- I am unclear why the method underpinning the qualitative analysis is presented in this paper but not the quantitative analysis. I can understand that each quantitative driven paper will likely have its own analytical plan, meaning this cannot be useful detailed herein. However, I would have thought this was also possible for the qual data, so to provide details for one but not the other in this paper feels rather unbalanced to me and I’d opt to provide neither herein.

**Discussion:**

- ‘for web’ – perhaps add in ‘samples’ before the references?
- It would be helpful to explain why not conducting f2f interviews was a drawback, since this is not necessarily the case for telephone interviews, which themselves have many advantages (e.g. better perceived anonymity, greater participant and researcher convenience etc.), which may also explain why video interviews are not preferred.

‘In conclusion, the Natsal-COVID Wave 1 study has enabled us to quantify and better understand the initial impacts of the COVID-19 pandemic on sexual behaviour and SRH in Britain following the start of the first national lockdown in the UK in March 2020.’

- Though I have no doubt that these data will allow this, you cannot conclude this from the information presented in this paper. Accordingly, I would recommend you focus your conclusion on the analysis presented herein; that it is possible to collect data using online panel sampling methods, which are convincingly comparable to representative samples conducted using offline methods (etc.).

Is the rationale for developing the new method (or application) clearly explained?

Yes

Is the description of the method technically sound?

Yes

Are the conclusions about the method and its performance adequately supported by the findings presented in the article?

Partly

If any results are presented, are all the source data underlying the results available to ensure full reproducibility?

No source data required

Are sufficient details provided to allow replication of the method development and its use by others?

Yes

Reviewer Expertise:

Sexual health, health psychology, sexual behaviour research, cross sectional longitudinal surveys, online & real world survey methodologies

I confirm that I have read this submission and believe that I have an appropriate level of expertise to confirm that it is of an acceptable scientific standard, however I have significant reservations, as outlined above.

# Response

**Published:** 2022-03-15

**Authors:** Emily Dema

**Affiliations:** University College London, UK

*Responses are italicised.*

 Approval Status: 

 The methods used are clearly and appropriately described within the paper and Wellcome Open Research is an appropriate forum for this paper. Accordingly, I approve this paper subject to very minor revisions, which I have detailed below. 

*RESPONSE: Thank you for these comments, which we have responded to below. We feel these have really helped to improve the paper.*

 Article Summary: 

 This study reports the methodology, quota sampling and weighting for an internet panel-based iteration of the decennial national probability Natsal (National Survey of Sexual Attitudes and Lifestyles) study, itself the world-leading study of sexual behaviours for a national population. C19 restrictions prevented the normal sampling methodology of Natsal during 2020; this paper critically discusses the new methodology developed to address this. 

 Specific questions for Article type: 

- Is the rationale for developing the new method (or application) clearly explained? 

Yes. This study reports the methodology, quota sampling and weighting for an internet panel-based iteration of the national probability Natsal study, itself the world-leading study of sexual behaviours for a national population. C19 restrictions prevented the normal sampling methodology of Natsal during 2020, so this paper is required to explain the new methodology developed. Moreover, this methodology represents the state of the art in terms of developing potentially representative national samples during C19, and so the rationale for developing this method is clear. It is also directly relevant to other situations where researchers need to assess the generalisability of their internet-based samples to the wider population. 

- Is the description of the method technically sound? 

Yes. The authors have provided extensive details around their method which are technically sound throughout and provide useful and clear critical reflection on their achievements and limitations. 

- Are sufficient details provided to allow replication of the method development and its use by others? 

Yes. The authors have provided a clear explanation of the methods they have used to develop their study, achieving an optimal balance of detail and brevity where appropriate, including referring the reader to existing documentation already available to enhance their description. There are some areas where a replicator may need additional details from the authors (e.g. the developmental history of specific survey questions) but these are best obtained from directly contacting the authors, rather than including all possible permutations in the paper. 

- If any results are presented, are all the source data underlying the results available to ensure full reproducibility? 

N/A. Study results are not presented other than the number of types of participants, by relevant characteristics, required to generate weighted data and check the representativeness of their samples against external datasets. 

- Are the conclusions about the method and its performance adequately supported by the findings presented in the article? 

Yes, all conclusions are highly appropriate, except for the final conclusion, which I have commented on below. In addition, I have made a few extra comments that the authors might find useful in enhancing their paper for clarity. 

 Peer Review Report: 

 Abstract: 

- It is unclear why qual participants were included so it would be helpful to briefly indicate the reason for this. No further comments by my side 

*RESPONSE: In the background we state: ‘We also undertook qualitative interviews to gain greater contextual understanding and to provide insights into the thoughts, feelings, and behaviours of participants reporting three types of experience in the survey, chosen for their public health importance.’ We feel this is complete and don’t propose to alter the manuscript.*

  

- ‘and inform policy’ – this paper, which focuses on reporting study methodology, does not provide any evidence about this issue so perhaps it should be deleted from this abstract. 

*RESPONSE: We will delete “and inform policy” from the abstract.*

 Key messages: 

 These are clear and appropriate. 

 Background: 

- A clear, balanced rationale has been provided for the importance of the Natsal-COVID study. 
- It would be helpful to specify the dates of wave 2 and the ‘types of experience’ used to sample qual participants. It would be useful to update on the report which was due in July 2021, if possible. 

*RESPONSE: After the sentence “We also undertook qualitative interviews to gain greater contextual understanding and to provide insights into the thoughts, feelings, and behaviours of participants reporting three types of experience in the survey, chosen for their public health importance”, we will add “The reported experiences were: sex with someone outside the household during lockdown; difficulty accessing SRH services; and relationship difficulties.” We have also specified the months for Wave 2 data collection (March-April 2021).*

  

- Figure 1 is helpful, but the ‘Natsal’ row needs greater explanation. Specifically, it is not immediately unclear what ‘to 1 yr prior to interview’ and ‘period of reporting’ mean. 

*RESPONSE: Participants were asked about behaviours and outcomes during the period from the start of lockdown (23 March 2020). We refer to this 4-month period as ‘period of reporting’. Some outcomes for the ‘past year’ were also asked. We have updated Figure 1 so that this is clearer.*

  

- It is important to clarify early on that Natsal 4 was planned for 2020 but paused due to the pandemic – perhaps in paragraph 2 of the background – which is why Natsal-COVID was conducted (if this is indeed the case). 

*RESPONSE: We will add the following sentence in paragraph 2. ““The pilot for the fourth decennial Natsal survey was paused until 2021 due to these restrictions, allowing the team to conduct the Natsal-COVID study.”*

 Sample Design: 

- A clear description of the target sample size, using design effect, power, change detected and risk ratio is provided for the overall sample size and the 18-29 boost sample. Although the process described is logical, I always think it is useful to refer readers to relevant texts/papers to confirm the literature underpinning these analyses (although I note that it is usual to miss this out). 

*RESPONSE: Thanks for the comment. Such papers will be referred to in subsequent work that arises from the Natsal-COVID study.*

  

- It might be helpful to clarify that the 2011 census is the most recently available in the UK at this time. 

*RESPONSE: We will update the sentence to the following: “The quotas for social grade used census data from 2011 (as mid-year or more recent estimates were not available for this measure).”*

   

- Is there a typo in the first sentence ‘and McNemar’s test’ 

*RESPONSE: This typo will be deleted.*

   

 Ethics: 

- These issues are clearly explained. Ethics obtained from U of G and LSHTM and therefore appropriate. 

 

 The Questionnaire: 

- Content is clearly summarized, with a convincing rationale. A good balance between brevity and depth has been achieved here and the actual survey is available online. It is always desirable for specific details of all survey questions’ development/origins to be available (and herein the C19 questions in particular). However, it is not feasible to provide such details for every item for such a large, detailed questionnaire. Therefore I agree with the level of detail provided herein and that further details should be restricted to future publications, where necessary. Perhaps the authors could suggest that further details about the origins/development of specific items could be provided upon request? 

*RESPONSE: The following sentence will be added. “For further information about the questionnaire, interested researchers may contact the Natsal team (natsal@ucl.ac.uk)”*

 Sample recruitment: 

- It would be helpful to modify the 2nd and 3rd sentences to clarify what the ‘online panels’ are; it is not actually made clear for folk who are not familiar with Ipsos MORI. It would be useful to know what/who the ‘six other panel providers’ were, for completeness. 

*RESPONSE: It is not possible to provide this level of detail because it is commercially sensitive information.*

   

- ‘Quota group’ – it might be helpful to signal that this issue is discussed in later in the paper. 

*RESPONSE: The following will be added after “quota group”: (described below)*

  

- ‘Median interview length’ – it would be helpful to replace the phrase ‘interview’ here with ‘survey’ (e.g. survey completion time’) as here I assume the authors are referring to the quant survey data completion and not the later qualitative interviews? Referring to both as interviews is likely to confuse these two different parts of the data collection. 

*RESPONSE: We will change “interview” to “survey”.*

 Gender: 

- Gender has been dealt with in an appropriate way herein, and clearly explained which is commendable. In addition, a reasonable proportion of trans folk have been included in the study which is a good achievement for this kind of general population survey. 

 

 Quota Filling: 

- It is important to have a couple of sentences to clarify what quotas were originally set up for the study as this is not clearly mentioned earlier. It might be useful to reference the technical reports mentioned later to help with this. Relaxation of quota sampling is clearly explained as is weighting. 

*RESPONSE: The sentence will be updated to include the weighting criteria as follows, “The initial weights (including gender, age, region, social grade, and ethnicity) did not include sexual identity…”*

 Representativeness: 

- This issue is clearly explained, and appropriately and comprehensively conducted with reference to appropriate external datasets. Over-representation of LGBTQI+ folk is to be expected given the focus on sexual health and sampling frame, though it might be worth clarifying why this is in the paper. The over-representation of folk reporting ‘fair’ as opposed to ‘very good’ health is to be expected during a global pandemic as are lower proportions reporting ‘sex in the past year’ after 4 months of lockdown and prohibited extra-domestic liaisons. Overall, a clear case is made for the representativeness of the sample given the potential for real behavioural changes engendered by the pandemic itself. 

 

 Qualitative sampling: 

- It might be useful to clearly locate this quota sampling as qualitative purposive quota sampling in the text. 

*RESPONSE: We have replaced ‘Quotas were used to:’ with ‘We used qualitative purposive sampling to’.*

  

- ‘women in group 2’ – I do not understand what being in ‘Group 2’ means? This appears in Box 2 but at this stage the reader has yet to be introduced to this information. Perhaps the first sentence of this section needs to clarify this. 

*RESPONSE:  We have reworded this sentence as follows to clarify groups: (group 1) sexual contact with someone living outside their household, (group 2) needing, but being unable to access SRH services, and (group 3) increased arguments and reduced support from their partner since lockdown.*

 Qualitative analysis: 

- I am unclear why the method underpinning the qualitative analysis is presented in this paper but not the quantitative analysis. I can understand that each quantitative driven paper will likely have its own analytical plan, meaning this cannot be useful detailed herein. However, I would have thought this was also possible for the qual data, so to provide details for one but not the other in this paper feels rather unbalanced to me and I’d opt to provide neither herein. 

*RESPONSE: We only provide very basic information on qualitative analysis that is common across all qualitative outputs. We believe this will be of interest to qualitative researchers. Given that the paper as a whole is unbalanced towards the quantitative survey, we would prefer to keep this in.*

 Discussion: 

- ‘for web’ – perhaps add in ‘samples’ before the references? 

*RESPONSE: The text has been updated.*

   

- It would be helpful to explain why not conducting f2f interviews was a drawback, since this is not necessarily the case for telephone interviews, which themselves have many advantages (e.g. better perceived anonymity, greater participant and researcher convenience etc.), which may also explain why video interviews are not preferred. 

*RESPONSE: In sexual health research, there are many advantages to f2f surveys. Response rates are much higher, we are able to randomly select one individual per address, interviewer rapport encourages participation and facilitates privacy, participant safety is maintained, questionnaire length can be longer, and biological sampling can be used with high success. F2f data collection would predominantly be self-completion on the interviewer’s laptop. The advantages you have listed for telephone interviews are actually much greater for f2f interviews with self-completion.*

 ‘In conclusion, the Natsal-COVID Wave 1 study has enabled us to quantify and better understand the initial impacts of the COVID-19 pandemic on sexual behaviour and SRH in Britain following the start of the first national lockdown in the UK in March 2020.’ 

- Though I have no doubt that these data will allow this, you cannot conclude this from the information presented in this paper. Accordingly, I would recommend you focus your conclusion on the analysis presented herein; that it is possible to collect data using online panel sampling methods, which are convincingly comparable to representative samples conducted using offline methods (etc.). 

*RESPONSE: We have updated the conclusion paragraph to focus on the analysis and quasi-representative nature of the sample, which can be analysed for broader SRH impact.*
